# Evaluation of the permeation of antineoplastic agents through medical gloves of varying materials and thickness and with varying surface treatments

**DOI:** 10.1186/s40780-017-0082-y

**Published:** 2017-05-02

**Authors:** Toyohito Oriyama, Takehito Yamamoto, Yoshitsugu Yanagihara, Katsuhiko Nara, Toshihide Abe, Katsuyoshi Nakajima, Takao Aoyama, Hiroshi Suzuki

**Affiliations:** 1 Department of Pharmacy, The University of Tokyo Hospital, Faculty of Medicine, The University of Tokyo, 7-3-1 Hongo, Bunkyo-ku, Tokyo, 113-8655 Japan; 2 Tokyo University of Science, Faculty of Pharmaceutical Sciences, 2641 Yamazaki, Noda, Chiba 278-8510 Japan; 3 The Education Center for Clinical Pharmacy, Graduate School of Pharmaceutical Sciences, The University of Tokyo, 7-3-1 Hongo, Bunkyo-ku, Tokyo, 113-0033 Japan

**Keywords:** Antineoplastic agents, Medical gloves, Permeability, Thickness, Surface treatment, Latex, Nitrile

## Abstract

**Background:**

Medical gloves are an important piece of personal protective equipment that prevents exposure to antineoplastic agents. The permeability of medical gloves to antineoplastic agents is a crucial factor in the appropriate selection of gloves. However, the relationship between glove permeability and material type, thickness, and surface treatment is poorly understood.

**Methods:**

A continuous flow in-line cell device was used for the evaluation of the permeation of five antineoplastic agents (etoposide, cyclophosphamide, doxorubicin hydrochloride, paclitaxel, and fluorouracil) through medical gloves. Medical gloves made of three types of materials (chlorinated latex, non-chlorinated latex, and nitrile) were subjected to a permeability test. The antineoplastic agents in test solutions were tested at the highest concentrations employed in general clinical practice. Then, the relationship between glove thickness and permeability was assessed using chlorinated latex gloves with thicknesses of 0.1, 0.15, 0.2, and 0.1 mm × 2 (to represent the practice of “double gloving”).

**Results:**

Only cyclophosphamide and fluorouracil showed detectable permeation through the tested latex gloves. The permeability of chlorinated latex was lower than that of non-chlorinated latex. Nitrile gloves showed no detectable permeability to any of the five antineoplastic agents tested. The permeability of chlorinated latex gloves depended on the thickness of the gloves; 0.1 mm × 2 (double gloving) exhibited the highest resistance to permeation by antineoplastic agents.

**Conclusions:**

The permeability of medical gloves was dependent on the type of material and the surface treatment and decreased as the thickness of the glove increased. The double glove was shown to prevent antineoplastic agent permeation more efficiently than did a single glove of the same total thickness. These results provided important information that will guide the appropriate selection of medical gloves.

## Background

It is well known that antineoplastic agents are potentially cytotoxic, mutagenic, teratogenic, and carcinogenic [1, 2]. Hence, healthcare professionals must take care to avoid unintended exposure during the handling process of these agents. The correct use of safety cabinets, closed-system transfer devices, and personal protective equipment (PPE), such as gloves, masks, and gowns, is essential to protect healthcare professionals from exposure to antineoplastic agents used in the clinical setting. However, several reports have indicated that healthcare professionals are exposed to antineoplastic agents despite the use of preventive devices and equipment, and may therefore have an increased risk of carcinogenesis [3–6]. To counter this situation, guidelines have recently been formulated to standardize the relevant procedures for clinical settings [7–9], which provide recommendations for the optimal workplace requirements, handling procedures to minimize the exposure to antineoplastic agents, and instructions for the proper use of PPEs.

Among the various items of PPE, medical gloves encounter the highest risk of exposure to antineoplastic agents during the handling process; thus, a selection of medical gloves with low permeability to antineoplastic agents are required to prevent healthcare professionals from unintended exposure. Owing to the importance of this requirement, the permeability of gloves to antineoplastic agents has been extensively investigated and some antineoplastic agents, such as cyclophosphamide (CPA) and carmustine, have been reported to penetrate through standard medical gloves [10]_._ Several studies have investigated the relationship between the type of materials, thickness of medical gloves, and permeability to antineoplastic agents. These studies showed that the permeability was dependent on the type of materials and thickness; however it was also shown that gloves made of the same material and thickness showed varying permeability depending on the brand [11], which suggested that manufacturing processes may alter the permeability of the gloves. Furthermore, gloves may receive surface treatment with chlorine or other chemicals to improve their fitting; the effects of such treatments on permeability have not been elucidated. Therefore, we chose to study the permeability of gloves that are made on the same manufacturing line, in addition to the possible effects of surface treatment.

In this study, we conducted the permeability tests using medical gloves made of latex (with and without surface treatment by chlorine) and nitrile to assess the effect of the types of materials and the thickness of gloves on the permeation of antineoplastic agents. Furthermore, we evaluated whether “double gloving” (i.e., wearing two sets of gloves), which is recommended in the guidelines for handling antineoplastic agents [8, 9, 12], had a preventive effect on permeation.

## Methods

### Test agents

Among the seven antineoplastic agents listed in protocol D6978-05 from the American Society of Testing and Materials (ASTM), the following five antineoplastic agents, which were available in Japan as intravenous formulations, were used in the permeability test: etoposide (ETP, Lastet® Injection, Nippon Kayaku Co., Ltd), CPA (Endoxan® for injection, Shionogi & Co., Ltd), doxorubicin (DXR) hydrochloride (ADRIACIN® Injection, Kyowa Hakko Kirin Co., Ltd), paclitaxel (PTX, TAXOL® INJECTION, Bristol-Myers Squibb), and fluorouracil (5FU, 5-FU Injection 250 Kyowa®, Kyowa Hakko Kirin Co., Ltd). For use in the tests, CPA and DXR hydrochloride were dissolved in normal saline, while the solutions of ETP, PTX, and 5FU were tested directly. The concentrations of solutions used for the permeability tests (C_test_), molecular weight (MW), and logarithm of the partition coefficient (logP) of the five selected antineoplastic agents are summarized in Table 1 [13–16].

**Table 1:** Antineoplastic agents used in this study

Antineoplastic agents	Brand name	Concentration [mg/mL]	MW^a^	logP^b^
Etoposide	Lastet® Inj. 100 mg/5 mL (Nippon Kayaku Co., Ltd)	20	588.6	0.6 [13]
Cyclophosphamide monohydrate	Endoxan® 100 mg (Shionogi & Co., Ltd)	20	261.1^c^	0.6 [13]
Doxorubicin hydrochloride	ADRIACIN® Injection 10 (Kyowa Hakko Kirin Co., Ltd)	10	543.5^d^	1.4 [14]
Paclitaxel	TAXOL® INJECTION 30 mg (Bristol-Myers Squibb)	6	853.9	3.7 [15]
Fluorouracil	5-FU Injection 250 Kyowa® (Kyowa Hakko Kirin Co., Ltd.)	50	130.8	−1.0 [16]

^a^molecular weight

^b^logarithm of Partition coefficient

^c^as anhydride

^d^as free base

The compounds of ETP, CPA, and 5FU were purchased from Sigma-Aldrich (St. Louis, MO, USA), and DXR hydrochloride and PTX were purchased from Wako Pure Chemical Industries, Ltd (Osaka, Japan). Gemcitabine hydrochloride was purchased from Toronto Research Chemicals Inc. (Toronto, Canada) and served as the internal standard for the 5FU assay. All other chemicals and organic solvents used in this study were of reagent or analytical grade.

### Medical gloves

Three types of gloves were used in this study: latex rubber with a non-chlorinated surface (latex A), latex rubber with a chlorinated surface (latex B), and nitrile rubber (nitrile). The glove thickness was approximately 0.1 mm for all materials.

The relationship between permeability and glove thickness was investigated using gloves made of latex B with different thicknesses (0.1, 0.15, and 0.2 mm). In addition, to evaluate the permeability of antineoplastic agents through double gloves, latex B gloves with a thickness of 0.1 mm were placed on top of each other (0.1 mm × 2) and subjected to the permeability test described below. All gloves used in this study were supplied by Okamoto Industries, Inc. (Tokyo, Japan) after inspection of thickness. The allowance range of thickness was set at ±0.03 mm from specified values.

### Permeability test

**Fig. 1:**
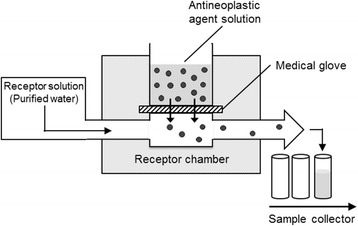
The schematic diagram of the flow in-line cell system

The permeability tests were performed according to methods described in the ASTM protocols D6978-05 and F739-07 [17, 18] (Fig. 1). In brief, medical gloves (contact area: 1 cm^2^) were placed in an ILC14 continuous flow in-line cell (PermeGear Inc., Hellertown, PA, USA), and the receptor solution (purified water) was pumped through the receptor chamber at a flow rate of 1 mL/h. The surface temperature of the glove was maintained at 27 °C throughout the experiment following the recommendation in F739-07 protocol [17]. Then, 1 mL antineoplastic agent solution was dropped onto the upper side of glove and the receptor solutions were collected for 0–15, 15–30, 30–60, 60–120, and 120–240 min after the addition of antineoplastic agent solution. After collection, specimens (0.2–0.5 mL aliquots) were transferred into the polypropylene sample tubes and stored at −80 °C until assay. Before use, the surface of the latex A gloves was washed with purified water to remove the surface powder and air-dried.

### Analytical procedure

The concentrations of antineoplastic agents in the specimens were measured by ultra-performance liquid chromatography-tandem mass spectroscopy (UPLC-MS/MS). The system consisted of an ACQUITY^TM^ binary solvent manager, ACQUITY^TM^ sample manager, and Quattro Premier XE triple quadrupole mass spectrometer (Waters Corp., Milford, MA, USA). The capillary voltage, ion source temperature, desolvation gas temperature, desolvation gas flow, and collision cell gas flow were set at 1 kV, 120 °C, 350 °C, 650 L/h, and 50 L/h, respectively. An ACQUITY^TM^ BEH AMIDE column (1.7 μm, 2.1 × 150 mm, Waters) was used for the 5FU assay and an ACQUITY^TM^ BEH Shield RP18 column (1.7 μm, 2.1 × 100 mm, Waters) was used for the measurement of other antineoplastic agents. During the assay, column temperature was kept at 40 °C using column oven built in the ACQUITY^TM^ sample manager. The details of the chromatographic conditions and MS/MS settings are summarized in Table 2. Before UPLC-MS/MS assay, the samples (100 μL) were spiked with 10 μL internal standard solution (5 μg/mL ETP for the CPA, DXR, and PTX assay; 5 μg/mL CPA for the ETP assay; 50 ng/mL of gemcitabine for the 5FU assay), vortex mixed for 1 min, and centrifuged (5 min, 4 °C, 20,000 *g*). Finally, 2 μL of clear supernatant was injected onto the UPLC-MS/MS system.

**Table 2:** UPLC-MS/MS conditions and settings

Analyte	Monitor ion [m/z]	Column^a^	Flow [mL/min]	Composition (solvent A/solvent B)^b^			LOQ [ng/mL]
ETP^c^	589.5 > 229.1	A	0.3	30/70 (isocratic)			50
CPA^d^	261.1 > 139.7	A	0.3	30/70 (isocratic)			40
DXR	544.4 > 397.2	A	0.3	0–3 3–4.5 4.5–5.5	min min min	90/10 90/10 → 10–90 90/10	50
PTX	854.6 > 286.0	A	0.3	0–3 3–4.5 4.5–5.5	min min min	70/30 70/30 → 10–90 70/30	200
5FU	128.6 > 85.6^e^	B	0.3	50/50 (isocratic)^g^			100
Gemcitabine^f^	264.1 > 111.6						-

^a^Column A, ACQUITY UPLC BEH® Shield RP18 (1.7 μm, 2.1 × 100 mm); column B, ACQUITY UPLC BEH® AMIDE (1.7 μm, 2.1 × 150 mm)

^b^Solvent A, 0.1% (v/v) formic acid in water; solvent B, 0.1% (v/v) formic acid in acetonitrile

^c^Also used as internal standard for CPA, DXR, and PTX

^d^Also used as internal standard for ETP

^e^5FU was detected by negative ion mode whereas the other compounds were detected by positive ion mode

^f^Internal standard for 5FU

^g^Water/acetonitrile = 50/50 (without 0.1% formic acid)

The limits of quantitation (LOQ) for the different compounds were as follows: ETP, 50 ng/mL; CPA, 40 ng/mL; DXR, 50 ng/mL; PTX, 200 ng/mL; and 5FU, 100 ng/mL. In these experimental conditions, the LOQ values of the tested antineoplastic agents corresponded to permeation rates (PR, described below) of 0.6–3.3 ng/min/cm^2^.

### Evaluation of permeability

The permeation of antineoplastic agents through medical gloves was evaluated using PR and breakthrough detection time (BTT) according to the ASTM protocol D6978-05. The PR value was calculated at each time point using the following formula [17]:

$$ \mathrm{P}\mathrm{R}\left(\mathrm{ng}/ \min /{\mathrm{cm}}^2\right)=\left(\mathrm{C}\times \mathrm{V}\right)/\mathrm{t}/\mathrm{S} $$

where C was the concentration of the antineoplastic agent in the receptor solution (ng/mL), V was the volume of collected receptor solution (mL), t was the exposure time (min), and S was the area of the glove surface that was exposed to the antineoplastic agent (1.0 cm^2^).

The first time point at which PR values exceeded 10 ng/min/cm^2^ was defined as the BTT (min) according to the ASTM D6978-05 protocol.

The cumulative permeated amount (X_cum_) was the total amount of antineoplastic agent recovered in the receptor solution until each time point: X_cum_ at time t is summation of the product of C and V in each sample fraction collected until time t.

To compare the permeation of CPA and 5FU in latex B gloves of varying thickness, we applied one-way ANOVA followed by a Student-Newman-Keuls test. Values of *P* < 0.05 were considered statistically significant.

## Results

### Permeability of antineoplastic agents through latex and nitrile gloves

**Fig. 2:**
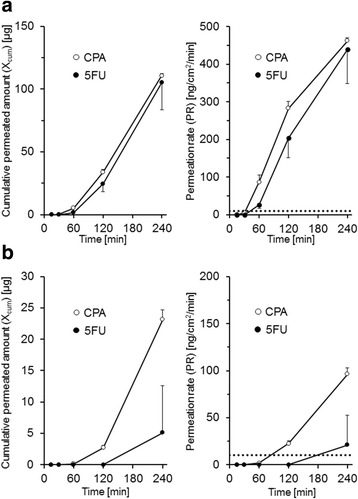
Permeation of CPA and 5FU through latex gloves with different surface treatments. The cumulative permeated amounts and PR values of CPA (*open circles*) and 5FU (*closed circles*) through latex A (panel **a**) and latex B (panel **b**) gloves are shown. The points and bars indicate the mean and SD, respectively (*n* = 3). The dotted line indicates the upper limit of the PR value in the ASTM guideline (10 ng/min/cm^2^). The concentrations of ETP, DXR, and PTX in the receptor solution were below the LOQ throughout the test period

The values for X_cum_ and PR for each antineoplastic agent at each time point are shown in Fig. 2. For latex A gloves, the values of X_cum_ and PR of CPA and 5FU increased in a time-dependent manner (Fig. 2a); the BTT of CPA and 5FU was 60 min. Latex B gloves were less permeable to CPA and 5FU than latex A gloves (Fig. 2b) and the BTTs of CPA and 5FU were 120 and 240 min, respectively. In contrast, ETP, DXR, and PTX showed no detectable permeation through both latex A and B within the testing time (240 min); hence, the BTTs were determined as >240 min.

For nitrile gloves, as none of the five tested antineoplastic agents showed detectable permeation at any time point, the BTTs were therefore evaluated as >240 min.

### Relationship between permeability and glove thickness

**Fig. 3:**
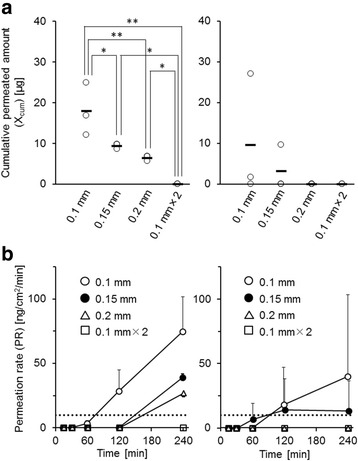
Relationship between permeation of antineoplastic agents and glove thickness. **a** The cumulative permeated amounts of CPA (*left panel*) and 5FU (*right panel*) through latex B gloves with thicknesses of 0.1, 0.15, 0.2, and 0.1 mm × 2 are shown. Open circles indicate individual data and bars indicate mean values. **b** The permeation rates (PR values) of CPA (*left panel*) and 5FU (*right panel*) through latex B gloves with thicknesses of 0.1 mm (*open circles*), 0.15 mm (*closed circles*), 0.2 mm (*open triangles*), and 0.1 mm × 2 (*open squares*) are shown. The points and bars indicate the mean and SD, respectively (*n* = 3). The dotted line indicates the upper limit of the PR value in the ASTM guideline (10 ng/min/cm^2^). *, **: *P* < 0.05 and *P* < 0.01, respectively (ANOVA followed by Student-Newman-Keuls test)

The values of X_cum_ and PR (at 240 min) for CPA and 5FU through latex B gloves of various thicknesses are shown in Fig. 3. For CPA, a higher PR and shorter BTT were observed with a reduced thickness of gloves (Fig. 3b and Table 3), which indicated that the permeability of CPA through latex B gloves was thickness dependent. The X_cum_ of CPA through latex B gloves at 240 min also showed a thickness dependent decrease, however the 0.1 mm × 2 (double glove) demonstrated a significantly lower X_cum_ than that of 0.2 mm glove, despite an equivalent total thickness (Fig. 3a).

**Table 3:** BTTs of antineoplastic agents for latex B gloves of varying thicknesses

Antineoplastic agent	BTT (min)			
	0.1 mm	0.15 mm	0.2 mm	0.1 mm × 2
ETP	>240	>240	>240	>240
CPA	120	240	240	>240
DXR	>240	>240	>240	>240
PTX	>240	>240	>240	>240
5FU	120	120	>240	>240

The X_cum_ and PR (at 240 min) of 5FU showed similar tendencies. The permeation of 5FU exceeded the ASTM standard in gloves with a thickness of 0.1 and 0.15 mm, but not of 0.2 mm or in the double glove, as shown in Fig. 3b. In accordance with these results, the X_cum_ of 5FU at 240 min tended to decrease as the thickness of gloves was increased; however, the differences were not statistically significant (Fig. 3a).

In contrast, ETP, DXR, and PTX exhibited no detectable permeation through latex B gloves of any thickness at any time point and the BTTs for these agents were >240 min (Table 3).

## Discussion

In this study, we performed permeability tests on medical gloves made on the same manufacturing line to assess the effects of material type, thickness, and surface treatment on the permeation of antineoplastic agents. The results of this study have several important implications. First, chlorine treatment of the glove surface affected its permeability to antineoplastic agents. Second, we confirmed that glove thickness was an important factor in determining permeability and showed that double gloving was a more effective barrier to the permeation of antineoplastic agents than a single glove of equivalent thickness.

Because previous studies indicated that MW and logP were important in determining the permeability of antineoplastic agents through medical gloves [11], we investigated the permeability using five antineoplastic agents with various MW and logP values (CPA, 5FU, DXR, ETP, and PTX) listed in the ASTM D6978-05 protocol. Although carmustine and thiotepa were also listed in the protocol as recommended compounds for permeability testing, we excluded these two agents for two reasons. First, intravenous formulations of carmustine and thiotepa are, at present, commercially unavailable in Japan. Second, the range of MW and logP values were 130.8–853.9 and -1.0–3.7, respectively, for the five antineoplastic agents (Table 1), and carmustine (MW = 214.1, logP = 1.53) [13] and thiotepa (MW = 189.2, and logP = 0.38) [19] were also within this range.

Among the five antineoplastic agents assessed in this study, only CPA and 5FU exhibited detectable permeation through gloves made of latex A and B. The PR values of CPA and 5FU through latex A gloves at the end of the testing time (240 min) were 4.79- and 20.7-fold higher, respectively, than those through latex B gloves (Fig. 2). The difference in permeability was likely caused by the chlorination of glove surface during the manufacturing process, because this was the only difference between the two materials. In everyday clinical settings, two types of latex gloves are commercially available: powdered and powder-free gloves (corresponding to latex A and B, respectively). Powder-free gloves are preferred when handling antineoplastic agents because the powder particles are considered to compromise sterile areas [7]. However, with regard to the permeability of the antineoplastic agents, direct comparisons between powdered and powder-free gloves have not been reported. In this study, we showed that powder-free (latex B) gloves were less permeable than powdered gloves (latex A) to CPA and 5FU, which indicated that the use of powder-free gloves was a reasonable method for reducing exposure to antineoplastic agents. Furthermore, our results also suggested that the manufacturing processes (especially surface treatments) should be considered when assessing the relationship between glove thickness and permeability. In contrast, none of the five tested antineoplastic agents exhibited detectable permeation through nitrile gloves with a thickness of 0.1 mm over a test time of 240 min. This observation supported the recommendations of several guidelines that suggest the use of nitrile gloves to prevent exposure to antineoplastic agents [8, 9, 12].

Next, the permeation of antineoplastic agents was assessed using latex B gloves of various thicknesses. The X_cum_ of CPA through latex B gloves within 240 min decreased in a thickness-dependent manner, with 0.1 mm × 2 (double glove) exhibiting the least permeability. Similar tendencies were observed for 5FU, although the differences among the thickness of gloves were not statistically significant (Fig. 3). These results confirmed previous reports that glove thickness was an influencing factor of the permeability of gloves to antineoplastic agents [11].

In addition, we observed a time-dependent increase in the PR values of CPA and 5FU (Figs. 2 and 3), which agreed with several previous reports [10, 11]. In our study conditions, C_test_ was much higher than those in receptor solution (>700 fold), and were regarded as stable during the permeability test. Therefore, the apparent permeation clearances (CL_P,app_, calculated by dividing PR by C_test_) were also observed to increase in a time-dependent manner, which indicated that the diffusion rate constants of antineoplastic agents within glove material were slower than those in the interface between the glove material and receptor solution. The diffusion kinetics demonstrated that PR was proportional to concentration of antineoplastic agents in the vicinity of interface between glove material and receptor solution (C_inter_), rather than C_test_. Assuming that the diffusion rate constant in glove material was lower than that at interface, C_inter_ would increase slowly after start of permeability test, and PR and CL_P,app_ would also increase in a time-dependent manner until C_inter_ and C_test_ reached the equilibrium state.

Theoretically, the PR values of antineoplastic agents were expected to be higher as logP was higher and MW was lower, because latex is a carbohydrate polymer and highly lipophilic. Indeed, a previous study examining the permeation of various antineoplastic agents has reported such a trend [11]. The results of our study were consistent with the report: The PR value (in a single 0.1 mm glove at 240 min) for CPA (74.8 ng/min/cm^2^) was higher than 5FU (40.0 ng/min/cm^2^), which has lower lipophilicity compared with CPA (the logP values for 5FU and CPA are −1.0 and 0.6, respectively). Because PR is also dependent on C_test_, we calculated the CL_P,app_ by dividing PR by C_test_ (20 mg/mL and 50 mg/mL for CPA and 5FU, respectively) to normalize the difference in C_test_ and evaluate the intrinsic permeability of antineoplastic agents through gloves. The calculated CL_P,app_ for CPA and 5FU was 3.7 μL/min/cm^2^ and 0.8 μL/min/cm^2^, respectively, and the CL_P,app_ of CPA was approximately 4.6-fold higher than that of 5FU. The difference appears to predominantly reflect the difference in logP between CPA and 5FU, because the magnitude of difference between CPA and 5FU is larger for logP compared with the MW. However, PTX showed no detectable permeation through medical gloves tested in this study although the logP of PTX is 3.7, the highest among the five antineoplastic agents tested. This discrepancy between logP and permeability can be explained by the large MW of PTX. The large MW of PTX (853.9) appears to reduce the diffusion rate constant in glove material and results in low permeability. The same explanation is also applicable for ETP (MW = 588.6), which showed no detectable permeation despite of the comparable logP (0.6) with CPA. Similarly, 5FU showed the detectable permeation despite of low logP (-1.0), and this observation appears to be attributed to the small MW of the compound (130.8). Further elucidation is required to confirm the exact effects of the logP and MW of antineoplastic agents on the permeation through medical gloves.

Interestingly, although the total thickness was equal in both conditions, the double glove (0.1 mm × 2) blocked the permeation of CPA more effectively compared to a single 0.2 mm glove (Fig. 3). BTTs for CPA were also longer in double glove than single 0.2 mm glove (>240 min vs 240 min, respectively) (Table 3). These results indicated that double gloving reduced the permeation of antineoplastic agents through a mechanism other than increased glove thickness, at least for CPA. Although double gloving is recommended in the ASHP guidelines on handling hazardous drugs and other guidelines [8, 9, 12] and is carried out in routine clinical settings, the information concerning the permeation of antineoplastic agents through double gloves is limited. To the best of our knowledge, this is the first report that quantitatively evaluates the permeation of antineoplastic agents through double gloves in comparison to a single glove with equivalent thickness. The underlying mechanism of this observation remains to be elucidated in future studies, we hypothesized the diffusion rate of CPA at the contact surface of the two gloves was slower than within glove material, and consequently attenuated the permeation of CPA through the double glove.

Finally, we considered the limitations of this study. First, the permeability of carmustine and thiotepa, which are recommended test compounds in the ASTM protocol, were not assessed in this study. Because carmustine (MW = 214.1, logP = 1.53) and thiothepa (MW = 189.2, logP = 0.38) have a similar MW and higher lipophilicity than 5FU (MW = 130.8, logP = −1.0), these agents may permeate through the medical gloves assessed in this study. However, as these two agents are unavailable as intravenous formulations in Japan, we believes that the permeability test using carmustine and thiothepa was not essential for the evaluation of medical gloves used in Japanese clinical settings. Second, all experiments in this study were conducted in static condition, which may not reflect the dynamic conditions (i.e., stretching or rubbing) to which the medical gloves are exposed in usual clinical workflow. In several previous studies [11, 20], permeability tests were conducted in dynamic conditions to estimate the maximum risk of permeation in real-life situations. However the exact effects of stretching or rubbing are not quantitatively determined because the permeability of same medical gloves in static conditions were not tested in these studies. Therefore, it is unclear whether our results are directly applicable to dynamic conditions, and future studies are needed to clarify the effects of stretching or rubbing on the permeability of medical gloves. The third limitation of this study is relatively short testing time. The PR values of CPA and 5FU increased in a time-dependent manner throughout the 240-min test period (Figs. 2 and 3), which indicated that the permeation of CPA and 5FU did not reached a steady state; we expect that PR values would increase with an extended testing time. In addition, it is possible that ETP, DXR, and PTX, which showed no detectable permeation within 240 min, exhibited a higher value of PR than the ASTM standard (10 ng/min/cm^2^) at later time points. However, because some guidelines recommend the frequent change of medical gloves when handling antineoplastic agents [21], healthcare professionals do not ordinarily use the same gloves over a 240-min period in the usual clinical workflow. Therefore, we believe that a test time of 240 min was sufficient for the evaluation of the permeation risk of antineoplastic agents.

## Conclusion

The permeation of five antineoplastic agents, ETP, CPA, DXR, PTX, and 5FU, through gloves was dependent on the glove material and surface treatment. In addition, the permeability of the gloves to antineoplastic agents decreased with an increase in glove thickness and double gloving was shown to efficiently reduce the antineoplastic agent permeation compared to single gloves with equivalent total thickness. Although further studies are needed to fully understand the mechanisms determining the permeation of antineoplastic agents through medical gloves, this study provides important information to facilitate the appropriate selection of medical gloves.

## Abbreviations

5FU: Fluorouracil
BTT: Breakthrough detection time
C: Concentration of antineoplastic agent in the receptor solution
C_inter_: Concentration of antineoplastic agent in vicinity of interface between glove material and receptor solution
CL_P,app_: Apparent permeation clearance of gloves
CPA: Cyclophosphamide
C_test_: Concentration of antineoplastic agent in the test solution
DXR: Doxorubicin
ETP: Etoposide
logP: logarithm of the partition coefficient
LOQ: Limit of quantitation
MS/MS: Tandem mass spectroscopy
PR: Permeation rate
PTX: Paclitaxel
S: Surface area of the glove exposed to an antineoplastic agent
t: Exposure time
UPLC: Ultraperformance liquid chromatography
V: Volume of collected receptor solution
X_cum_: Cumulative amount of antineoplastic agents permeated through glove
